# Supplementary material for: Stress and benefits of video calling for people with autism spectrum disorders
Source: PLoS One. 2023 Apr 20;18(4):e0283526. doi: 10.1371/journal.pone.0283526 (PMC10118183; doi:10.1371/journal.pone.0283526)
Supplement: Attachment — Submitted filename: rebuttal letter0308.docx [file pone.0283526.s002.docx]

**Rebuttal letter**

We thank the reviewers for their valuable comments that have greatly improved our paper.

**Comment:**

３．Have the authors made all data underlying the findings in their manuscript fully available?

**Response:** We have added a Minimal Underlying dataset file to the Data Availability statement.

**Comment:** In methodology there was not mention about age limits. Age is an important indicator for coping any new application. In different age group person's ability to minimise stress at different level, like at early age group ASD people could cope early. Time of screen time also an important issue which author mentioned in limitation.

**Response:** Thank you for pointing this out. We have changed the following text in “Limitations” from (p. 30, lines 407-410):

Third, the frequency of video calling may have confounded the results. In particular, the results of video calling fatigue may differ depending on the call frequency [11]. In the future, analyzing the differences in experience according to frequency of use would be desirable.

to

Third, the age of participants and frequency of video calling use may confounded the results of the study. Younger age is a factor that increases the frequency of video calling use [29], and furthermore, frequency of use affects fatigue on video calling [11]. Therefore, the confounding of participants’ age may create a bias in the frequency of video calling use and change the perception of stress in video calling. In the future, analyzing the differences in experience according to age and frequency of video calling use.

Ｗe have also added the following reference.

29. Brown G, Greenfield PM. Staying connected during stay-at-home: Communication with family and friends and its association with well-being. Hum Behav Emerging Technol. 2021;3(1): 147-156. doi: 10.1002/hbe2.246.

We wish to thank the Reviewer again for his or her valuable comments.
